# Quorum sensing communication between bacteria and human cells: signals, targets, and functions

**DOI:** 10.3389/fpls.2014.00309

**Published:** 2014-06-26

**Authors:** Angelika Holm, Elena Vikström

**Affiliations:** Division of Medical Microbiology, Department of Clinical and Experimental Medicine, Faculty of Health Sciences, Linköping UniversityLinköping, Sweden

**Keywords:** bacteria–host cell interaction, quorum sensing, *Pseudomonas aeruginosa*, *N*-acyl homoserine lactones, epithelial cells, innate immune cells, neutrophils, macrophages

## Abstract

Both direct and long-range interactions between pathogenic *Pseudomonas aeruginosa* bacteria and their eukaryotic hosts are important in the outcome of infections. For cell-to-cell communication, these bacteria employ the quorum sensing (QS) system to pass on information of the density of the bacterial population and collectively switch on virulence factor production, biofilm formation, and resistance development. Thus, QS allows bacteria to behave as a community to perform tasks which would be impossible for individual cells, e.g., to overcome defense and immune systems and establish infections in higher organisms. This review highlights these aspects of QS and our own recent research on how *P. aeruginosa* communicates with human cells using the small QS signal molecules *N*-acyl homoserine lactones (AHL). We focus on how this conversation changes the behavior and function of neutrophils, macrophages, and epithelial cells and on how the signaling machinery in human cells responsible for the recognition of AHL. Understanding the bacteria–host relationships at both cellular and molecular levels is essential for the identification of new targets and for the development of novel strategies to fight bacterial infections in the future.

## INTRODUCTION

*Pseudomonas aeruginosa* is an environmentally highly adaptable Gram-negative bacterium that infects different host species, including higher plants, invertebrates, and vertebrates. In humans, it elicits acute and chronic infections, typically in critically ill patients having compromised epithelial barriers and immune system or the genetic disorder cystic fibrosis. The outcome of infections and establishment of disease depends on both host defence and bacterial capacities. The latter include its autonomic efficiency to grow, divide, and adapt to the environment, and the ability to sense, and communicate with their neighbors in the population to accomplish cooperative activities, e.g., biofilm formation and production of virulence factors. To do this, *P. aeruginosa* uses a mechanisms of cell-to-cell communication called quorum sensing (QS). It allows the bacteria to recognize the population density by sensing and measuring the accumulation of specific small signal molecules that members of the community secrete. When the population density is high, the amount of accumulated signals in the environment is accordingly sufficient to activate signaling pathways that alter bacterial gene expression and activate cooperative responses (Rutherford and Bassler, 2012; Schuster et al., 2013; Fazli et al., 2014).

## *P. aeruginosa* QS CONTROL OF VIRULENCE AND BIOFILM FORMATION

Being equipped with a relatively large genome,* P. aeruginosa* harbors three distinct but subordinated QS systems: two of LuxI/LuxR-type and a third called the *Pseudomonas* quinolone signal (PQS) system. The two LuxI/LuxR-type systems are *N*-acylhomoserine lactone (AHL) dependent. In the first, the LuxI homolog LasI produces a freely diffusible* N*-3-oxo-dodecanoyl-L-homoserine lactone (3O-C_12_-HSL) that is detected by the LuxR homolog cytoplasmic receptor LasR (More et al., 1996; Parsek et al., 1999). In the second, the LuxI homolog RhlI synthesizes another AHL,* N*-butyryl-L-homoserine lactone (C_4_-HSL) that binds to the cytoplasmic receptor RhlR (Ochsner et al., 1994; Pearson et al., 1995); LasR and RhlR are cognate transcriptional regulators. Together, the AHL–LuxR complexes of both circuits control the activation of more than 300 genes in the *P. aeruginosa* genome. Many of these genes code for production of extracellular products that may be considered as virulence factors, because they can damage host tissues and promote infection, and inflammation. These virulence factors include exotoxin A, elastase, proteases, pyocyanin, lectins, and toxins (Gambello and Iglewski, 1991; Toder et al., 1991; Gambello et al., 1993; Schuster et al., 2003). *P. aeruginosa* uses the third PQS system to control cooperative responses and gene expression of rhamnolipid, a critical biosurfactant in the late stage of biofilm formation (Ochsner et al., 1994; de Kievit, 2009). The signal molecules of this system are bicyclic compounds, 2-alkyl-4(1H)-quinolones (PQS), produced by PqsABCDH and recognized by the receptor PqsR (Deziel et al., 2004; Diggle et al., 2007). Several of PQS can act not only as a QS signals, but also possess antimicrobial, anticancer, or antiallergenic activities. Together with periplasmic components, outer membrane proteins, phospholipids, toxins, lipopolysaccharide (LPS), and DNA, PQS are typically packed into spherical 50–250 nm membrane vesicles that *P. aeruginosa* secrete and deliver to the environment. In this way, vesicles have a role in communication and competion in microbial communities and with host cells (Heeb et al., 2011; Tashiro et al., 2013). In other cases, *P. aeruginosa* can directly convey its products to other cells using the type VI secretion system. In addition, bacteria possesses an intracellular orphan receptor QscR, a LuxR homolog (Lintz et al., 2011) that can bind to 3O-C_12_-HSL (Oinuma and Greenberg, 2011). This natural target forms dimers with other receptors, i.e., LasR and RhlR, making them inactive and thereby repressing LasRI- and RhlRI-dependent genes leading to prevention of aberrant QS responses before the bacteria reach a quorum in a community (Ledgham et al., 2003). Furthermore, the LasR-3O-C_12_-HSL, RhlR-C_4_-HSL, and PQS-PqsR complexes target the regulation of *lasI, rhlI, pqsH,* and *pqsR,* which creates an autoinducing feed-forward loop and establishes the tightness and subordination between all three QS systems (Seed et al., 1995; Latifi et al., 1996; Deziel et al., 2004; Xiao et al., 2006; Diggle et al., 2007). Thus, with optimal precision *P. aeruginosa* QS system directly or indirectly controls the expression of more than 10% of genes for multiple virulence factors, secondary metabolites, swarming motility, and biofilm development (Schuster and Greenberg, 2006; Wagner and Iglewski, 2008; Williams and Camara, 2009).

## QS-DRIVEN INTER-KINGDOM SIGNALING

Prokaryotes and eukaryotes have coexisted for many years, during which time they have been exposed to the signals produced and released by the other. The organisms of two kingdoms have also learnt to sense their various molecules including QS signals to influence gene expression and behavior in a process called inter-kingdom signaling (Pacheco and Sperandio, 2009; Gonzalez and Venturi, 2013).

The QS signal molecule AHL affects mammalian host cells and its signaling pathways; this was shown using both *in vivo* and *in vitro* models for immune cells, fibroblasts, and epithelial cells. For the biological activity of AHL, a long acyl chain with more than 10 C-atoms, an intact homoserine lactone ring, and oxo- or hydroxyl substitutions are important. AHL triggers and acts through multiple signaling pathways, e.g., calcium mobilization, activation of Rho GTPases, MAPK, and NFκB that control diverse functions and behaviors in host cells, like cytoskeleton remodeling, chemotaxis, migration, phagocytosis, epithelial barrier function, differentiation, proliferation, apoptosis, and production of immune mediators. This topic has been thoroughly investigated by many research groups during the last decade and extensively reviewed recently (Williams and Camara, 2009; Jarosz et al., 2011; Teplitski et al., 2011).

Many organisms, including bacteria, fungi, plants, and mammalian can disturb and inactivate AHL by enzymes in a way called quorum quenching (Czajkowski and Jafra, 2009; Chen et al., 2013). Humans have also developed an ability to destroy AHL via a class of quorum quenching enzymes called paraoxonases (Amara et al., 2011).

Here, we will further focus on mostly our research on how bacterial QS conversation changes the behavior and function of human neutrophils, macrophages, and epithelial cells, and the signaling responsible for the recognition of AHL.

## EPITHELIAL BARRIER INTEGRITY AND 3O-C_12_-HSL

**FIGURE 1:**
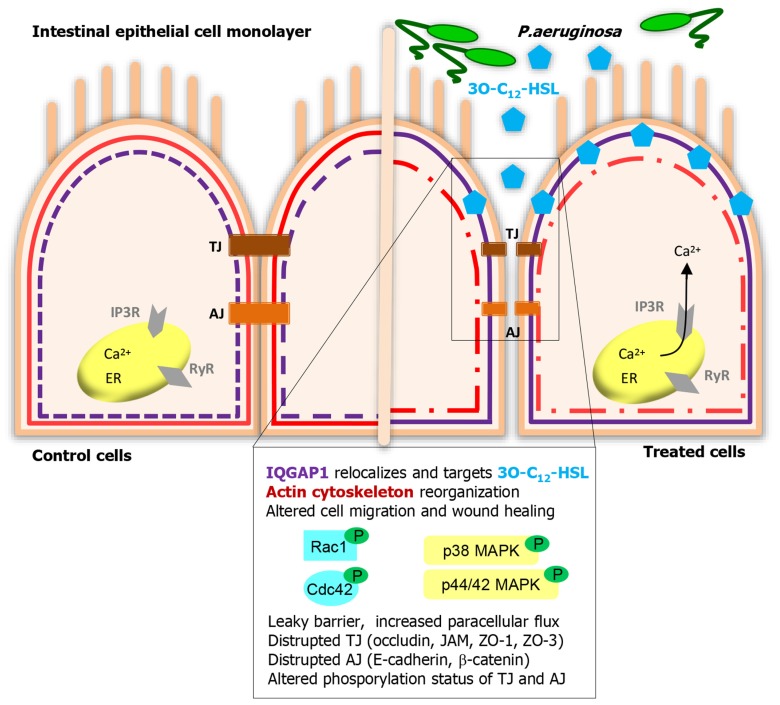
**Human intestinal epithelial cells listen to and act on *Pseudomonas aeruginosa* 3O-C**_**12**_-HSL signaling. IQGAP1 is a novel membrane-associated target for 3O-C_12_-HSL that helps human epithelial cell recognize and response to bacterial QS signaling. Further, 3O-C_12_-HSL triggers multiple signaling pathways, those include calcium mobilization, activation of Rho GTPases Rac1/Cdc42, MAPK, protein kinases, and protein phosphotases. These can control diverse functions and behaviors in epithelial cells, like cytoskeleton remodeling, migration, wound healing, barrier integrity, and paracellular permeability.

Epithelial cells are positioned strategically to provide both physical and immune barriers to pathogens and other environmental agents. The physical barrier is created by epithelial cell-to-cell junctions that prevent for instance invasion of pathogens and food constituents. The junctions are multiprotein associations of transmembrane proteins connected to cytoplasmic plaque proteins and the actin cytoskeleton (Van Itallie and Anderson, 2004; Balda and Matter, 2008; Furuse, 2010; Ivanov et al., 2010; Capaldo et al., 2014). In a model of polarized epithelial cells, 3O-C_12_-HSL alters their barrier integrity (**Figure 1**), as evidenced by decreased transepithelial electrical resistance and increased paracellular flux of different-sized dextrans (Vikstrom et al., 2006). The cell junction complexes occludin–ZO-1, JAM–ZO-1 and E-cadherin–β-catenin were disrupted and the expression and distribution of proteins were affected (Vikstrom et al., 2006, 2009, 2010). Some junctional cytoplasmic proteins play a scaffolding role in linking the actin cytoskeleton and helping to recruit an array of signaling pathways, for example the MAPK cascade molecules, protein kinases, and protein phosphatases (Balda and Matter, 2009; Rodgers and Fanning, 2011). Both p38 and p42/44 MAPK are involved in the 3O-C_12_-HSL-induced leaky barrier (Vikstrom et al., 2006). Moreover, the disrupted cell junction associations and enhanced paracellular permeability are paralleled by alterations in the phosphorylation status of TJ and AJ proteins (**Figure 1**; Vikstrom et al., 2009, 2010). Ca^2^^+^ is another important component in the regulating of immune and physical barriers of the epithelium. Mucosal epithelial cells are as other cells, equipped with Ca^2^^+^-dependent signaling which allows them to initiate immune response to bacteria and their products. The participation of Ca^2^^+^ as a second messenger is thus vital to many physiological processes of the epithelia including response to bacteria (Vandenbroucke et al., 2008; Varani, 2011). 3O-C_12_-HSL can mobilize intracellular calcium through influx from surrounding and release from thapsigargin-sensitive stores via inositol 1,4,5-triphosphate receptors, IP_3_R (**Figure 1**; Vikstrom et al., 2010). These receptors are based in the endoplasmatic reticulum (ER) and regulated by their messengers IP_3_ (Ivanova et al., 2014). Together with ER, the actin cytoskeleton is also critically involved in Ca^2^^+^ storage and release as well as in the regulation of store-coupled Ca^2^^+^ entry (Lange and Gartzke, 2006).

## 3O-C_12_-HSL-MEDIATED EPITHELIAL MIGRATION AND WOUND HEALING

Establishing and contributing to both physical and immune barriers, the epithelial cells also have to be constantly renewed and prepared to move. After injury, caused by for example oxidative stress, inflammation and infection, the epithelium undergo a wound-healing process. This is dependent on the balance of migration, proliferation, and differentiation of the cells within the wound area (Sturm and Dignass, 2008). Restitution of the epithelium requires extensive reorganization of the cytoskeleton and cellular junctions, regulated by the Rho family of small GTPases, like Rho, Rac, and Cdc42 (Kjoller and Hall, 1999; Evers et al., 2000). 3O-C_12_-HSL modulates epithelial cell migration in a dose- and time-dependent manner (Karlsson et al., 2012b) inducing remodeling of cytoskeletal actin (Vikstrom et al., 2006). The upstream effectors of this, and thereby regulators of cell shape and motility are the previously mentioned Rho family GTPases Rac1 and Cdc42. The Rho GTPases cycle between an active and inactive status by binding GTP and by hydrolysis of GTP to GDP, acting as molecular “on-off” switch (Wennerberg and Der, 2004). The signaling can also be modulated by their phosphorylation state via AKT1 kinase (Kwon et al., 2000). Together with the effect of 3O-C_12_-HSL on cell migration, low doses of 3O-C_12_-HSL over shorter time spans initiated phosphorylation of Rac1/Cdc42, whereas high concentrations rapidly decrease the level of phosphorylated Rac/Cdc42 (Karlsson et al., 2012b). Taken together, 3O-C_12_-HSL can alter barrier functions and migration in epithelial cells (**Figure 1**).

The immune barrier of epithelial cells is potentiated through detection of antigens and rapid signaling to recruit phagocytes to the site of infection and tissue damage. They paracellulary transmigrate across the epithelium, and cell junction protein JAM mediate this process as it is also receptor for leukocyte integrins (Zen and Parkos, 2003). Phagocytes, like neutrophils and macrophages, are key players in the innate immune defenses, providing protection from invading bacteria and tissue damage. Bacterial QS conversation may change their behavior and function and the signaling responsible for the sensing of AHL.

## AHL AS A STRONG CHEMOATTRACTANT FOR NEUTROPHILS

**FIGURE 2:**
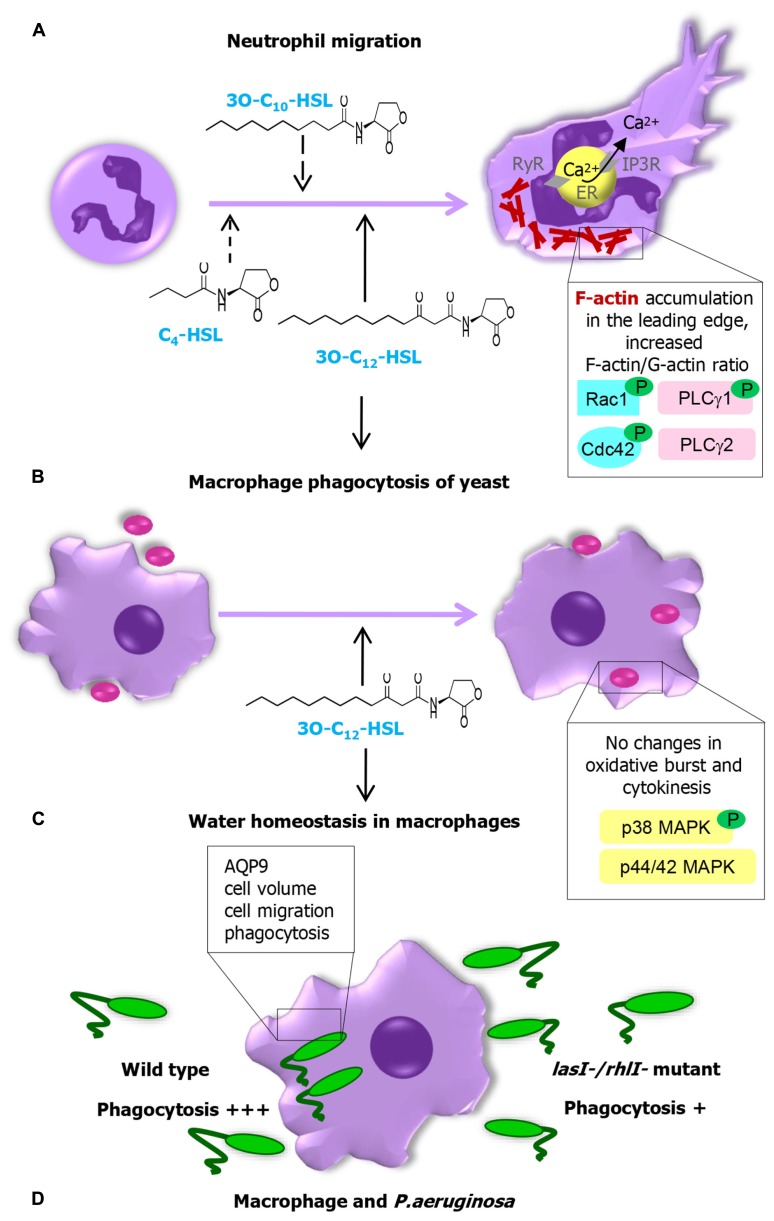
***Pseudomonas aeruginosa* AHL changes the behavior and function of human innate immune cells and the signaling machinery responsible for the recognition of AHL. (A)** Long chain AHLs are strong chemoattractants for neutrophils. Chemotaxis and migration towards these AHLs are paralleled by cytoskeletal F-actin accumulation in the leading edge and increased F-actin-to-G-actin ratio via activation of small GTPases Rac1/Cdc42, phosphorylation of PLCγ1 and mobilization of calcium via IP_3_R at ER. **(B)** 3O-C_12_-HSL and macrophage activity. 3O-C_12_-HSL increases phagocytic capacity of macrophages via the p38, but not p42/44 MAPK, having no influence on their oxidative metabolism, or production of cytokines. **(C)** 3O-C_12_-HSL-mediated water homeostasis in macrophages. **(D)** Macrophage phagocytosis of *P. aeruginosa* wild type and *lasI-/rhlI-* mutant lacking production of QS molecules 3O-C_12_-HSL and C_4_-HSL.

Neutrophils are small and rapidly moving phagocytes with a short life span in circulation, always appearing at the early onset of infection. They sense the bacteria very well, which gives them a head start in controlling inflammation, infection, and biofilm formation. Following activation by bacterial products or immune stimuli, they execute specialized functions of chemotaxis, phagocytosis, and generation of reactive oxygen species (ROS). 3-oxo-C_12_-HSL and 3-oxo-C_10_-HSL, but not C_4_-HSL, can act as a strong chemoattractants for human neutrophils and induce their migration to the site of AHL in a dose-dependent manner (Zimmermann et al., 2006; Karlsson et al., 2012a). This put long chain AHL in a position among potent *bona fide* chemoattractants, such as chemokines, cytokines (IL-8 and GM-CSF), leukotriene B_4_, platelet activating factor, products from bacteria (formylated peptides and LPS), signals from dying cells (such as TNF-α), although higher molar concentrations of AHL were required (**Figure 2A**). Chemotaxis and migration towards AHL are paralleled by cytoskeletal F-actin accumulation in the leading edge of neutrophils, by increased F-actin-to-G-actin ratio and via activation of Rho GTPases Rac1 and Cdc42 (Karlsson et al., 2012a). Rac1 regulates the formation of lamellipodia protrusions and membrane ruffles, and Cdc42 triggers filopodial extensions (Kjoller and Hall, 1999; Evers et al., 2000). Two long acyl chain AHL with 10 and 12 C-atoms activated phospholipase Cγ1 and mobilized intracellular calcium via the IP_3_R at endoplasmic reticulum, whereas an AHL with a short acyl chain failed to do this (Karlsson et al., 2012a; **Figure 2A**). Thus, recognition of AHL QS signals by neutrophils may play a critical role in their recruitment during infections and early stage of biofilm formation.

## AHL AND MACROPHAGE ACTIVITY

Other important players in the innate immune system are macrophages, cells differentiated from monocytes in tissues and stimulated by neutrophils to come to the site of infection after them. Macrophages harbor the characteristics of innate immune cells and traits that initiate mechanisms of adaptive immunity. In response to microbial antigens, they can display strong phagocytic activity, generation of “killer” anti-microbial ROS, NO, “tissue repair” ornithine, pro-inflammatory cytokines stimulating other immune cells to respond to pathogens. In a macrophage phagocytosis model with *Saccharomyces cerevisiae* as preys, 3O-C_12_-HSL increased phagocytic capacity via the p38, but not the p42/44 MAPK signaling pathway (**Figure 2B**). It had no influence on macrophages oxidative metabolism, the level of ROS or production of cytokines (Vikstrom et al., 2005). In *P. aeruginosa*, two functional QS genes, *lasI,* and* rhlI*, which are responsible for synthesis of signal molecules 3O-C_12_-HSL and C_4_-HSL strongly contribute to effective macrophage phagocytosis of this pathogen. AQP9 controls cell migration by accumulation in membrane protrusions and domains preceding their expansions (Karlsson et al., 2011, 2013a, b). This trigger actin cytoskeleton to be remodeled and further regulate macrophage shape, migration, and phagocytosis of microorganisms (**Figure 2D**). 3O-C_12_-HSL caused a rapid and prolonged cell-volume increase controlled by AQP9 in human macrophages (**Figure 2C**), which can be a danger signal and protection mechanism (Hoffmann et al., 2009; Compan et al., 2012). AQP9 is involved in *Escherichia coli* LPS-enhanced brain water content and blood-brain barrier permeability (Wang et al., 2009) and has been identified as a one of markers of chronic inflammation in patients with psoriasis, rheumatoid arthritis, and inflammatory bowel disease (Mesko et al., 2010).

## HUMAN CELL TARGETS FOR 3O-C_12_-HSL

Identifying targets for AHL allows better understanding of QS communication during host–bacteria interactions. The recognition of 3O-C_12_-HSL by mammalian cells probably does not rely on pattern-recognition receptors (PRRs) that usually sense invariant microbial motifs (PAMPs, pathogen-associated molecular patterns) present on or shed from bacteria (LPS, lipoteichoic acid, flagellin, and DNA). The canonical class of PRRs, the membrane-bound toll-like receptors (TLRs) located on immune cells, do not interact with 3O-C_12_-HSL (Kravchenko et al., 2006). Still, as for TLR activation, AHL can trigger and act through multiple signaling pathways, which include calcium signaling, activation of Rho GTPases, MAPK, and transcription factor NFκB that control expression of pro-inflammatory mediators, cytokines, chemokines, enzymes, and interferones (Smith et al., 2002; Shiner et al., 2006; Kravchenko et al., 2008; Mayer et al., 2011; Karlsson et al., 2012a; Glucksam-Galnoy et al., 2013). These mediators are involved in the coordination of innate immune response and recruit effector cells of the adaptive immune system to the site of the infection to combat the invading bacteria.

During the recognition of 3O-C_12_-HSL by mammalian cells, this lipophilic molecule with a long acyl chain and an intact homoserine lactone ring may interact directly with phospholipids in model membrane systems and in T-cell membranes (Davis et al., 2010). On entering host cells (Shiner et al., 2004; Ritchie et al., 2007), 3O-C_12_-HSL can utilize intracellular nuclear peroxisome proliferator-activated receptors (PPAR) to affect NF-κB signaling (Jahoor et al., 2008; Cooley et al., 2010). The binding of 3O-C_12_-HSL to nuclear PPAR does not exclude the existence of cell surface or membrane-associated receptors, which after binding to 3O-C_12_-HSL likely help phosphorylate phospholipase C and evoke an increase in intracellular calcium (Shiner et al., 2006; Davis et al., 2010; Karlsson et al., 2012a).

Several groups have designed probes and affinity matrixes which could be utilized to detect the mammalian target of 3O-C_12_-HSL (Dubinsky et al., 2009; Garner et al., 2011; Praneenararat et al., 2011; Dubinsky et al., 2013). Using a biotin-based 3O-C_12_-HSL probe, LC-MS/MS, and super-resolution microscopy, the IQ-motif-containing GTPase-activating protein IQGAP1 was identified as a putative human target for 3O-C_12_-HSL in epithelial cells (**Figure 1**; Karlsson et al., 2012b). IQGAP1 contains multiple domains for binding other proteins and localizes in the leading edge of migrating cells (Briggs and Sacks, 2003; Noritake et al., 2004; Bensenor et al., 2007). It directly interacts with and stabilizes the Rho-family GTPases, Rac1, and Cdc42 in their GTP-bound state (Swart-Mataraza et al., 2002; Briggs and Sacks, 2003), playing an essential role in cell shape, vesicle trafficking, and directional migration (Bensenor et al., 2007). It likely mediates these processes through its other domains, linking it to actin, myosin, β-catenin, E-cadherin, calmodulin, and MAPK (Noritake et al., 2005; Brandt and Grosse, 2007), which allows it to function as a true scaffolding protein (**Figure 1**). The interaction of 3O-C_12_-HSL with the cell membrane, diffusion and entering into the cytoplasm, targeting of IQGAP1 and PPAR do not exclude each other. It has, for example been shown that different types of lipids, such as chemoattractant leukotriene B_4_, can bind to both the cell surface receptor LTB4, and nuclear PPAR.

## CONCLUDING REMARKS

During the last decade of research remarkable insight has been gained into the mechanisms of bacterial QS communication and that *P. aeruginosa* 3O-C_12_-HSL plays at least two distinct roles. Besides regulating bacterial social behavior and offering density-dependent fitness advantages, expression of virulence factors, and biofilm development in bacteria, it also plays a crucial role in the behavior of eukaryotic host cells regulating various vital functions. Moreover, as QS circuits often control virulence and biofilm, there is a high interest in interfering with QS as a new strategy to overcome infectious diseases and biofilm formation (Amara et al., 2011; Heeb et al., 2011; Jakobsen et al., 2013; LaSarre and Federle, 2013).

## Conflict of Interest Statement

The authors are not aware of any affiliations, memberships, patents, copyrights, funding, payment, service or other activities or relationships that might be perceived as an affecting the objectivity of this review.

## References

[B1] Amara N. Krom B. P. Kaufmann G. F. Meijler M. M. (2011). Macromolecular inhibition of quorum sensing: enzymes, antibodies, and beyond. *Chem. Rev.* 111 195–208 10.1021/cr100101c 21087050

[B2] Balda M. S. Matter K. (2008). Tight junctions at a glance. *J. Cell Sci.* 121(Pt22) 3677–3682 10.1242/jcs.023887 18987354

[B3] Balda M. S. Matter K. (2009). Tight junctions and the regulation of gene expression. *Biochim. Biophys. Acta* 1788 761–767 10.1016/j.bbamem.2008.11.024 19121284

[B4] Bensenor L. B. Kan H. M. Wang N. Wallrabe H. Davidson L. A. Cai Y. (2007). IQGAP1 regulates cell motility by linking growth factor signaling to actin assembly. *J. Cell Sci.* 120(Pt 4) 658–669 10.1242/jcs.03376 17264147

[B5] Brandt D. T. Grosse R. (2007). Get to grips: steering local actin dynamics with IQGAPs. *EMBO Rep.* 8 1019–1023 10.1038/sj.embor.7401089 17972901 PMC2247391

[B6] Briggs M. W. Sacks D. B. (2003). IQGAP1 as signal integrator: Ca^2^^+^, calmodulin, Cdc42 and the cytoskeleton. *FEBS Lett.* 542 7–11 10.1016/S0014-5793(03)00333-8 12729888

[B7] Capaldo C. T. Farkas A. E. Nusrat A. (2014). Epithelial adhesive junctions. *F1000Prime Rep.* 6:1 10.12703/P6-1 PMC3883420 24592313

[B8] Chen F. Gao Y. Chen X. Yu Z. Li X. (2013). Quorum quenching enzymes and their application in degrading signal molecules to block quorum sensing-dependent infection. *Int. J. Mol. Sci.* 14 17477–17500 10.3390/ijms140917477 24065091 PMC3794736

[B9] Compan V. Baroja-Mazo A. Lopez-Castejon G. Gomez A. I. Martinez C. M. Angosto D. (2012). Cell volume regulation modulates NLRP3 inflammasome activation. *Immunity* 37 487–500 10.1016/j.immuni.2012.06.013 22981536

[B10] Cooley M. A. Whittall C. Rolph M. S. (2010). *Pseudomonas* signal molecule 3-oxo-C12-homoserine lactone interferes with binding of rosiglitazone to human PPARgamma. *Microbes Infect.* 12 231–237 10.1016/j.micinf.2009.12.009 20074659

[B11] Czajkowski R. Jafra S. (2009). Quenching of acyl-homoserine lactone-dependent quorum sensing by enzymatic disruption of signal molecules. *Acta Biochim. Pol.* 56 1–16 19287806

[B12] Davis B. Jensen M. R. Williams P O’Shea P. (2010). The interaction of N-acylhomoserine lactone quorum sensing signaling molecules with biological membranes: implications for inter-kingdom signaling. *PLoS ONE* 5:e13522 10.1371/journal.pone.0013522 PMC2958149 20975958

[B13] de Kievit T. R. (2009). Quorum sensing in *Pseudomonas aeruginosa* biofilms. *Environ. Microbiol.* 11 279–288 10.1111/j.1462-2920.2008.01792.x 19196266

[B14] Deziel E. Lepine F. Milot S. He J. Mindrinos M. N. Tompkins R. G. (2004). Analysis of *Pseudomonas aeruginosa* 4-hydroxy-2-alkylquinolines (HAQs) reveals a role for 4-hydroxy-2-heptylquinoline in cell-to-cell communication. *Proc. Natl. Acad. Sci. U.S.A.* 101 1339–1344 10.1073/pnas.0307694100 14739337 PMC337054

[B15] Diggle S. P. Matthijs S. Wright V. J. Fletcher M. P. Chhabra S. R. Lamont I. L. (2007). The *Pseudomonas aeruginosa* 4-quinolone signal molecules HHQ and PQS play multifunctional roles in quorum sensing and iron entrapment. *Chem. Biol.* 14 87–96 10.1016/j.chembiol.2006.11.014 17254955

[B16] Dubinsky L. Delago A. Amara N. Krief P. Rayo J. Zor T. (2013). Species selective diazirine positioning in tag-free photoactive quorum sensing probes. *Chem. Commun. (Camb.)* 49 5826–5828 10.1039/c3cc43092h 23702727 PMC3723129

[B17] Dubinsky L. Jarosz L. M. Amara N. Krief P. Kravchenko V. V. Krom B. P. (2009). Synthesis and validation of a probe to identify quorum sensing receptors. *Chem. Commun. (Camb.)* 47 7378–7380 10.1039/b917507e 20024234

[B18] Evers E. E. Zondag G. C. Malliri A. Price L. S. ten Klooster J. P. van der Kammen R. A. (2000). Rho family proteins in cell adhesion and cell migration. *Eur. J. Cancer* 36 1269–1274 10.1016/S0959-8049(00)00091-5 10882865

[B19] Fazli M. Almblad H. Rybtke M. L. Givskov M. Eberl L. Tolker-Nielsen T. (2014). Regulation of biofilm formation in *Pseudomonas* and *Burkholderia* species. *Environ. Microbiol*. 10.1111/1462-2920.12448 [Epub ahead of print] 24592823

[B20] Furuse M. (2010). Molecular basis of the core structure of tight junctions. *Cold Spring Harb. Perspect. Biol.* 2:a002907 10.1101/cshperspect.a002907 PMC2827901 20182608

[B21] Gambello M. J. Iglewski B. H. (1991). Cloning and characterization of the *Pseudomonas aeruginosa* lasR gene, a transcriptional activator of elastase expression. *J. Bacteriol.* 173 3000–3009 1902216 10.1128/jb.173.9.3000-3009.1991 PMC207884

[B22] Gambello M. J. Kaye S. Iglewski B. H. (1993). LasR of *Pseudomonas aeruginosa* is a transcriptional activator of the alkaline protease gene (apr) and an enhancer of exotoxin A expression. *Infect. Immun.* 61 1180–1184 8454322 10.1128/iai.61.4.1180-1184.1993 PMC281346

[B23] Garner A. L. Yu J. Struss A. K. Lowery C. A. Zhu J. Kim S. K. (2011). Synthesis of ‘clickable’ acylhomoserine lactone quorum sensing probes: unanticipated effects on mammalian cell activation. *Bioorg. Med. Chem. Lett.* 21 2702–2705 10.1016/j.bmcl.2010.11.122 21190852 PMC3081916

[B24] Glucksam-Galnoy Y. Sananes R. Silberstein N. Krief P. Kravchenko V. V. Meijler M. M. (2013). The bacterial quorum-sensing signal molecule N-3-oxo-dodecanoyl-L-homoserine lactone reciprocally modulates pro- and anti-inflammatory cytokines in activated macrophages. *J. Immunol.* 191 337–344 10.4049/jimmunol.1300368 23720811 PMC3691282

[B25] Gonzalez J. F. Venturi V. (2013). A novel widespread interkingdom signaling circuit. *Trends Plant Sci.* 18 167–174 10.1016/j.tplants.2012.09.007 23089307

[B26] Heeb S. Fletcher M. P. Chhabra S. R. Diggle S. P. Williams P. Camara M. (2011). Quinolones: from antibiotics to autoinducers. *FEMS Microbiol. Rev.* 35 247–274 10.1111/j.1574-6976.2010.00247.x 20738404 PMC3053476

[B27] Hoffmann E. K. Lambert I. H. Pedersen S. F. (2009). Physiology of cell volume regulation in vertebrates. *Physiol. Rev.* 89 193–277 10.1152/physrev.00037.2007 19126758

[B28] Ivanov A. I. Parkos C. A. Nusrat A. (2010). Cytoskeletal regulation of epithelial barrier function during inflammation. *Am. J. Pathol.* 177 512–524 10.2353/ajpath.2010.100168 20581053 PMC2913378

[B29] Ivanova H. Vervliet T. Missiaen L. Parys J. B. De Smedt H. Bultynck G. (2014). Inositol 1,4,5-trisphosphate receptor-isoform diversity in cell death and survival. *Biochim. Biophys. Acta* 10.1016/j.bbamcr.2014.03.007 [Epub ahead of print] 24642269

[B30] Jahoor A. Patel R. Bryan A. Do C. Krier J. Watters C. (2008). Peroxisome proliferator-activated receptors mediate host cell proinflammatory responses to *Pseudomonas aeruginosa* autoinducer. *J. Bacteriol.* 190 4408–4415 10.1128/JB.01444-07 18178738 PMC2446782

[B31] Jakobsen T. H. Bjarnsholt T. Jensen P. O. Givskov M. Hoiby N. (2013). Targeting quorum sensing in *Pseudomonas aeruginosa* biofilms: current and emerging inhibitors. *Future Microbiol.* 8 901–921 10.2217/fmb.13.57 23841636

[B32] Jarosz L. M. Ovchinnikova E. S. Meijler M. M. Krom B. P. (2011). Microbial spy games and host response: roles of a *Pseudomonas aeruginosa* small molecule in communication with other species. *PLoS Pathog.* 7:e1002312 10.1371/journal.ppat.1002312 PMC3219704 22114549

[B33] Karlsson T. Bolshakova A. Magalhaes M. A. Loitto V. M. Magnusson K. E. (2013a). Fluxes of water through aquaporin 9 weaken membrane-cytoskeleton anchorage and promote formation of membrane protrusions. *PLoS ONE* 8:e59901 10.1371/journal.pone.0059901 PMC3616121 23573219

[B34] Karlsson T. Lagerholm B. C. Vikstrom E. Loitto V. M. Magnusson K. E. (2013b). Water fluxes through aquaporin-9 prime epithelial cells for rapid wound healing. *Biochem. Biophys. Res. Commun.* 430 993–998 10.1016/j.bbrc.2012.11.125 23261438

[B35] Karlsson T. Glogauer M. Ellen R. P. Loitto V. M. Magnusson K. E. Magalhaes M. A. (2011). Aquaporin 9 phosphorylation mediates membrane localization and neutrophil polarization. *J. Leukoc. Biol.* 90 963–973 10.1189/jlb.0910540 21873454

[B36] Karlsson T. Musse F. Magnusson K. E. Vikstrom E. (2012a). N-Acylhomoserine lactones are potent neutrophil chemoattractants that act via calcium mobilization and actin remodeling. *J. Leukoc. Biol.* 91 15–26 10.1189/jlb.0111034 21807742

[B37] Karlsson T. Turkina M. V. Yakymenko O. Magnusson K. E. Vikstrom E. (2012b). The *Pseudomonas aeruginosa* N-acylhomoserine lactone quorum sensing molecules target IQGAP1 and modulate epithelial cell migration. *PLoS Pathog.* 8:e1002953 10.1371/journal.ppat.1002953 PMC3469656 23071436

[B38] Kjoller L. Hall A. (1999). Signaling to Rho GTPases. *Exp. Cell Res.* 253 166–179 10.1006/excr.1999.4674 10579921

[B39] Kravchenko V. V. Kaufmann G. F. Mathison J. C. Scott D. A. Katz A. Z. Grauer D. C. (2008). Modulation of gene expression via disruption of NF-kappaB signaling by a bacterial small molecule. *Science* 321 259–263 10.1126/science.1156499 18566250

[B40] Kravchenko V. V. Kaufmann G. F. Mathison J. C. Scott D. A. Katz A. Z. Wood M. R. (2006). N-(3-oxo-acyl)homoserine lactones signal cell activation through a mechanism distinct from the canonical pathogen-associated molecular pattern recognition receptor pathways. *J. Biol. Chem.* 281 28822–28830 10.1074/jbc.M606613200 16893899

[B41] Kwon T. Kwon D. Y. Chun J. Kim J. H. Kang S. S. (2000). Akt protein kinase inhibits Rac1-GTP binding through phosphorylation at serine 71 of Rac1. *J. Biol. Chem.* 275 423–428 10.1074/jbc.275.1.423 10617634

[B42] Lange K. Gartzke J. (2006). F-actin-based Ca signaling-a critical comparison with the current concept of Ca signaling. *J. Cell. Physiol.* 209 270–287 10.1002/jcp.20717 16823881

[B43] LaSarre B. Federle M. J. (2013). Exploiting quorum sensing to confuse bacterial pathogens. *Microbiol. Mol. Biol. Rev.* 77 73–111 10.1128/MMBR.00046-12 23471618 PMC3591984

[B44] Latifi A. Foglino M. Tanaka K. Williams P. Lazdunski A. (1996). A hierarchical quorum-sensing cascade in *Pseudomonas aeruginosa* links the transcriptional activators LasR and RhIR (VsmR) to expression of the stationary-phase sigma factor RpoS. *Mol. Microbiol.* 21 1137–1146 10.1046/j.1365-2958.1996.00063.x 8898383

[B45] Ledgham F. Ventre I. Soscia C. Foglino M. Sturgis J. N. Lazdunski A. (2003). Interactions of the quorum sensing regulator QscR: interaction with itself and the other regulators of *Pseudomonas aeruginosa* LasR and RhlR. *Mol. Microbiol.* 48 199–210 10.1046/j.1365-2958.2003.03423.x 12657055

[B46] Lintz M. J. Oinuma K. Wysoczynski C. L. Greenberg E. P. Churchill M. E. (2011). Crystal structure of QscR, a *Pseudomonas aeruginosa* quorum sensing signal receptor. *Proc. Natl. Acad. Sci. U.S.A.* 108 15763–15768 10.1073/pnas.1112398108 21911405 PMC3179110

[B47] Mayer M. L. Sheridan J. A. Blohmke C. J. Turvey S. E. Hancock R. E. (2011). The *Pseudomonas aeruginosa* autoinducer 3O-C12 homoserine lactone provokes hyperinflammatory responses from cystic fibrosis airway epithelial cells. *PLoS ONE* 6:e16246 10.1371/journal.pone.0016246 PMC3031552 21305014

[B48] Mesko B. Poliska S. Szegedi A. Szekanecz Z. Palatka K. Papp M. (2010). Peripheral blood gene expression patterns discriminate among chronic inflammatory diseases and healthy controls and identify novel targets. *BMC Med. Genomics* 3:15 10.1186/1755-8794-3-15 PMC2874757 20444268

[B49] More M. I. Finger L. D. Stryker J. L. Fuqua C. Eberhard A. Winans S. C. (1996). Enzymatic synthesis of a quorum-sensing autoinducer through use of defined substrates. *Science* 272 1655–1658 10.1126/science.272.5268.1655 8658141

[B50] Noritake J. Fukata M. Sato K. Nakagawa M. Watanabe T. Izumi N. (2004). Positive role of IQGAP1, an effector of Rac1, in actin-meshwork formation at sites of cell-cell contact. *Mol. Biol. Cell* 15 1065–1076 10.1091/mbc.E03-08-0582 14699063 PMC363077

[B51] Noritake J. Watanabe T. Sato K. Wang S. Kaibuchi K. (2005). IQGAP1: a key regulator of adhesion and migration. *J. Cell Sci.* 118(Pt 10) 2085–2092 10.1242/jcs.02379 15890984

[B52] Ochsner U. A. Koch A. K. Fiechter A. Reiser J. (1994). Isolation and characterization of a regulatory gene affecting rhamnolipid biosurfactant synthesis in *Pseudomonas aeruginosa*. *J. Bacteriol.* 176 2044–2054 8144472 10.1128/jb.176.7.2044-2054.1994 PMC205310

[B53] Oinuma K. Greenberg E. P. (2011). Acyl-homoserine lactone binding to and stability of the orphan *Pseudomonas aeruginosa* quorum-sensing signal receptor QscR. *J. Bacteriol.* 193 421–428 10.1128/JB.01041-10 21097632 PMC3019841

[B54] Pacheco A. R. Sperandio V. (2009). Inter-kingdom signaling: chemical language between bacteria and host. *Curr. Opin. Microbiol.* 12 192–198 10.1016/j.mib.2009.01.006 19318290 PMC4852728

[B55] Parsek M. R. Val D. L. Hanzelka B. L. Cronan J. E. Jr. Greenberg E. P. (1999). Acyl homoserine-lactone quorum-sensing signal generation. *Proc. Natl. Acad. Sci. U.S.A.* 96 4360–4365 10.1073/pnas.96.8.4360 10200267 PMC16337

[B56] Pearson J. P. Passador L. Iglewski B. H. Greenberg E. P. (1995). A second N-acylhomoserine lactone signal produced by *Pseudomonas aeruginosa*. *Proc. Natl. Acad. Sci. U.S.A.* 92 1490–1494 10.1073/pnas.92.5.1490 7878006 PMC42545

[B57] Praneenararat T. Beary T. M. Breitbach A. S. Blackwell H. E. (2011). Synthesis and application of an N-acylated l-homoserine lactone derivatized affinity matrix for the isolation of quorum sensing signal receptors. *Bioorg. Med. Chem. Lett.* 21 5054–5057 10.1016/j.bmcl.2011.04.062 21592793 PMC3513337

[B58] Ritchie A. J. Whittall C. Lazenby J. J. Chhabra S. R. Pritchard D. I. Cooley M. A. (2007). The immunomodulatory *Pseudomonas aeruginosa* signalling molecule N-(3-oxododecanoyl)-L-homoserine lactone enters mammalian cells in an unregulated fashion. *Immunol. Cell Biol.* 85 596–602 10.1038/sj.icb.7100090 17607318

[B59] Rodgers L. S. Fanning A. S. (2011). Regulation of epithelial permeability by the actin cytoskeleton. *Cytoskeleton (Hoboken)* 68 653–660 10.1002/cm.20547 22083950 PMC3310364

[B60] Rutherford S. T. Bassler B. L. (2012). Bacterial quorum sensing: its role in virulence and possibilities for its control. *Cold Spring Harb. Perspect. Med* 2:a012427 10.1101/cshperspect.a012427 PMC3543102 23125205

[B61] Schuster M. Greenberg E. P. (2006). A network of networks: quorum-sensing gene regulation in *Pseudomonas aeruginosa*. *Int. J. Med. Microbiol.* 296 73–81 10.1016/j.ijmm.2006.01.036 16476569

[B62] Schuster M. Lostroh C. P. Ogi T. Greenberg E. P. (2003). Identification, timing, and signal specificity of *Pseudomonas aeruginosa* quorum-controlled genes: a transcriptome analysis. *J. Bacteriol.* 185 2066–2079 10.1128/JB.185.7.2066-2079.2003 12644476 PMC151497

[B63] Schuster M. Sexton D. J. Diggle S. P. Greenberg E. P. (2013). Acyl-homoserine lactone quorum sensing: from evolution to application. *Annu. Rev. Microbiol.* 67 43–63 10.1146/annurev-micro-092412-155635 23682605

[B64] Seed P. C. Passador L. Iglewski B. H. (1995). Activation of the *Pseudomonas aeruginosa* lasI gene by LasR and the *Pseudomonas* autoinducer PAI: an autoinduction regulatory hierarchy. *J. Bacteriol.* 177 654–659 7836299 10.1128/jb.177.3.654-659.1995 PMC176640

[B65] Shiner E. K. Reddy S. Timmons C. Li G. Williams S. C. Rumbaugh K. P. (2004). Construction of a bacterial autoinducer detection system in mammalian cells. *Biol. Proced. Online* 6 268–276 10.1251/bpo98 15630481 PMC539822

[B66] Shiner E. K. Terentyev D. Bryan A. Sennoune S. Martinez-Zaguilan R. Li G. (2006). *Pseudomonas aeruginosa* autoinducer modulates host cell responses through calcium signalling. *Cell Microbiol.* 8 1601–1610 10.1111/j.1462-5822.2006.00734.x 16984415

[B67] Smith R. S. Kelly R. Iglewski B. H. Phipps R. P. (2002). The *Pseudomonas* autoinducer N-(3-oxododecanoyl) homoserine lactone induces cyclooxygenase-2 and prostaglandin E2 production in human lung fibroblasts: implications for inflammation. *J. Immunol.* 169 2636–2642 10.4049/jimmunol.169.5.2636 12193735

[B68] Sturm A. Dignass A. U. (2008). Epithelial restitution and wound healing in inflammatory bowel disease. *World J. Gastroenterol.* 14 348–353 10.3748/wjg.14.348 18200658 PMC2679124

[B69] Swart-Mataraza J. M. Li Z. Sacks D. B. (2002). IQGAP1 is a component of Cdc42 signaling to the cytoskeleton. *J. Biol. Chem.* 277 24753–24763 10.1074/jbc.M111165200 11948177

[B70] Tashiro Y. Yawata Y. Toyofuku M. Uchiyama H. Nomura N. (2013). Interspecies interaction between *Pseudomonas aeruginosa* and other microorganisms. *Microbes Environ.* 28 13–24 10.1264/jsme2.ME12167 23363620 PMC4070684

[B71] Teplitski M. Mathesius U. Rumbaugh K. P. (2011). Perception and degradation of N-acyl homoserine lactone quorum sensing signals by mammalian and plant cells. *Chem. Rev.* 111 100–116 10.1021/cr100045m 20536120

[B72] Toder D. S. Gambello M. J. Iglewski B. H. (1991). *Pseudomonas aeruginosa* LasA: a second elastase under the transcriptional control of lasR. *Mol. Microbiol.* 5 2003–2010 10.1111/j.1365-2958.1991.tb00822.x 1766376

[B73] Van Itallie C. M. Anderson J. M. (2004). The molecular physiology of tight junction pores. *Physiology (Bethesda)* 19 331–338 10.1152/physiol.00027.2004 15546850

[B74] Vandenbroucke E. Mehta D. Minshall R. Malik A. B. (2008). Regulation of endothelial junctional permeability. *Ann. N. Y. Acad. Sci.* 1123 134–145 10.1196/annals.1420.016 18375586

[B75] Varani J. (2011). Calcium, calcium-sensing receptor and growth control in the colonic mucosa. *Histol. Histopathol.* 26 769–779 21472691 10.14670/hh-26.769 PMC4806791

[B76] Vikstrom E. Bui L. Konradsson P. Magnusson K. E. (2009). The junctional integrity of epithelial cells is modulated by *Pseudomonas aeruginosa* quorum sensing molecule through phosphorylation-dependent mechanisms. *Exp. Cell Res.* 315 313–326 10.1016/j.yexcr.2008.10.044 19038248

[B77] Vikstrom E. Bui L. Konradsson P. Magnusson K. E. (2010). Role of calcium signalling and phosphorylations in disruption of the epithelial junctions by *Pseudomonas aeruginosa* quorum sensing molecule. *Eur. J. Cell Biol.* 89 584–597 10.1016/j.ejcb.2010.03.002 20434232

[B78] Vikstrom E. Magnusson K. E. Pivoriunas A. (2005). The *Pseudomonas aeruginosa* quorum-sensing molecule N-(3-oxododecanoyl)-L-homoserine lactone stimulates phagocytic activity in human macrophages through the p38 MAPK pathway. *Microbes Infect.* 7 1512–1518 10.1016/j.micinf.2005.05.012 16039899

[B79] Vikstrom E. Tafazoli F. Magnusson K. E. (2006). *Pseudomonas aeruginosa* quorum sensing molecule N-(3 oxododecanoyl)-l-homoserine lactone disrupts epithelial barrier integrity of Caco-2 cells. *FEBS Lett.* 580 6921–6928 10.1016/j.febslet.2006.11.057 17157842

[B80] Wagner V. E. Iglewski B. H. (2008). P. aeruginosa Biofilms in CF Infection. *Clin. Rev. Allergy Immunol.* 35 124–134 10.1007/s12016-008-8079-9 18509765

[B81] Wang H. Jin R. Tian P. Zhuo Z. (2009). Enhanced expression of aquaporin-9 in rat brain edema induced by bacterial lipopolysaccharides. *J. Huazhong Univ. Sci. Technolog. Med. Sci.* 29 150–155 10.1007/s11596-009-0203-4 19399395

[B82] Wennerberg K. Der C. J. (2004). Rho–family GTPases: it’s not only Rac and Rho (and I like it). *J. Cell Sci.* 117(Pt 8) 1301–1312 10.1242/jcs.01118 15020670

[B83] Williams P. Camara M. (2009). Quorum sensing and environmental adaptation in *Pseudomonas aeruginosa*: a tale of regulatory networks and multifunctional signal molecules. *Curr. Opin. Microbiol.* 12 182–191 10.1016/j.mib.2009.01.005 19249239

[B84] Xiao G. He J. Rahme L. G. (2006). Mutation analysis of the *Pseudomonas aeruginosa* mvfR and pqsABCDE gene promoters demonstrates complex quorum-sensing circuitry. *Microbiology* 152(Pt 6) 1679–1686 10.1099/mic.0.28605-0 16735731

[B85] Zen K. Parkos C. A. (2003). Leukocyte-epithelial interactions. *Curr. Opin. Cell Biol.* 15 557–564 10.1016/S0955-0674(03)00103-0 14519390

[B86] Zimmermann S. Wagner C. Muller W. Brenner-Weiss G. Hug F. Prior B. (2006). Induction of neutrophil chemotaxis by the quorum-sensing molecule N-(3-oxododecanoyl)-L-homoserine lactone. *Infect. Immun*. 74 5687–5692 10.1128/IAI.01940-05 16988244 PMC1594900

